# Functional similarity analysis of human virus-encoded miRNAs

**DOI:** 10.1186/2043-9113-1-15

**Published:** 2011-05-19

**Authors:** Guangchuang Yu, Qing-Yu He

**Affiliations:** 1 Institute of Life and Health Engineering and National Engineering Research Center of Genetic Medicine, Jinan University, Guangzhou 510632, China

## Abstract

miRNAs are a class of small RNAs that regulate gene expression via RNA silencing machinery. Some viruses also encode miRNAs, contributing to the complex virus-host interactions. A better understanding of viral miRNA functions would be useful in designing new preventive strategies for treating diseases induced by viruses. To meet the challenge for how viruses module host gene expression by their encoded miRNAs, we measured the functional similarities among human viral miRNAs by using a method we reported previously. Higher order functions regulated by viral miRNAs were also identified by KEGG pathway analysis on their targets. Our study demonstrated the biological processes involved in virus-host interactions via viral miRNAs. Phylogenetic analysis suggested that viral miRNAs have distinct evolution rates compared with their corresponding genome.

## Introduction

miRNAs, about 22 nucleotides in length, constitute a large family of non-coding RNAs that regulate gene expression posttranscriptionally, leading their target mRNAs to direct destructive cleavage or translational repression by base pairing with the 3' untranslated regions (3' UTRs). miRNA-mediated regulation plays crucial roles in a wide spectrum of biological processes, including proliferation [1], apoptosis [2], development [3], immune system regulation [4], and oncogenesis [5].

Recent discoveries on viral miRNAs, mostly in herpesvirus family [6], threw lights on a new level of cross-talk between virus and host in viral infections and pathogenesis [7]. Viral miRNAs have been reported to participate in immune evasion by directly down-regulating host immune defence genes, and even to cooperate with viral proteins to target the same process [8]. The combination of protein-mediated and miRNA-mediated regulations forms an intricate strategy for viruses to resist host defence system and thus increase the opportunities of their survival.

The research on viral miRNAs is still far from exhausted, with many unknown miRNA functions yet to be discovered. miRNA identification using computational tools is the most widely used method. In contrast to most eukaryotic miRNAs, virus-encoded miRNAs do not have homologs in other viral genomes or in the genome of the human host [6], and thus are difficult to be identified using existing miRNA gene prediction tools. Cloning and sequencing small RNA libraries to identify and characterize miRNAs is the basic method for miRNA discovery, since computationally predicted miRNAs should also be confirmed by experimental methods. Reverse ligation-mediated RT-PCR [9] is a widely used method in the identification of mature miRNAs and has been used to detect maturely processed MuHV-4 miRNAs [10]. Experimental validation is still a barrier in miRNA identification, especially in host cells infected by viruses. Currently, only a small fraction of viral miRNAs has been identified, and the functions of most of these viral miRNAs remain unknown. To bridge the gap in understanding the targets regulated by these virus-encoded miRNAs, we used computational method to predict host targets of viral miRNAs and measured their functional similarities to reveal the interspecies cross-talk between virus and host by viral miRNAs.

## Materials and methods

### Host target gene prediction of viral miRNAs

In order to determine how viruses reshape the physiological states of human cells by their encoded miRNAs, we first predicted host genes targeted by viral miRNAs. We collected viral miRNAs encoded by BK polyomavirus (BKV), Epstein-Barr virus (EBV), human cytomegalovirus (HCMV), human immunodeficiency virus 1 (HIV1), human herpesvirus 1 (HSV1), human herpesvirus 2 (HSV2), and Kaposi's sarcoma-associated herpesvirus (KSHV). Viral miRNA sequences were retrieved from miRBase [11] release 16 (Sep 2010). We extracted 3' UTR sequences in a single FASTA format file from human genome (version 18) that was downloaded from UCSC [12].

Host target genes of virus-encoded miRNAs were predicted by the algorithm of Probability of Interaction by Target Accessibility (PITA) that computes the difference between the free energy gained from the formation of the miRNA-mRNA duplex and the energetic cost of unpairing the mRNA to make it accessible to the miRNA [13]. We chose PITA for viral miRNA target prediction because it had been demonstrated to reach high accuracy, and more importantly, it takes advantage of the target accessibility but not conservation information to reduce false positive. Conservation information, which was used by most of other methods, is not suitable for predicting target genes of the less evolutionarily conserved viral miRNAs [6]. We used a flank of 3 upstream and 15 downstream nucleotides when performing prediction, since miRNA-mRNA interaction requires unpairing of bases flanking the targets. To reduce false positive, the prediction results were narrowed down by the criteria of 7-8 bases seed length, with no G:U wobble or loops, no mismatch, and ∆∆G < -20 kcal/mol.

### Functional similarity measurement of viral miRNAs

We have previously proposed a method for systematic study of functional similarities among miRNAs by using their target genes Gene Ontology (GO) semantic similarities [14]. As described in our previous study, the functional similarity of human miRNAs, obtained by our method, showed positive correlation with expression similarity, and the clustering results derived from the functional similarity were coherent with biological knowledge in many aspects including disease association, genome conservation, and the cross-talk between hosts and viruses [14]. The method is reliable to calculate functional similarities and sensible to cluster miRNAs, and thus can be used to predict novel miRNA functions.

Here, we applied our method to measure functional similarities among viral miRNAs. As suggested in our previous study [14], the measurement was fundamentally based on host target genes of viral miRNAs. Biological process ontology was used to annotate target genes, and Wang's method [15] was used to calculate semantic similarity. Semantic similarity calculation was implemented by our in-house developed R package GOSemSim [16].

Similarity scores were then analyzed by R package pvclust [17], which used multi-scale bootstrap re-sampling to evaluate the uncertainty of cluster analysis. The agglomerative method, average linkage, was used, and 10,000 bootstrap replications were run. All clusters were extracted with approximately unbiased (AU) *p*-value > 0.95, meaning that the hypothesis with "the cluster does not exist" is rejected with significance level of 0.05.

### GO enrichment analysis of significant clusters

The common biological processes regulated by these significant miRNA clusters were evaluated by GOstats [18] with *p *< 0.001. GOstats using hypergeometric model to assess whether the number of selected genes associated with the GO term is larger than expected. This method had been used to predict the functions of miRNAs [14] and can be used to provide biological insights of viral strategies.

### KEGG enrichment analysis of genes targeted by viral miRNAs

In order to uncover higher order functions of how viruses transform cellular states by their encoded miRNAs, we adopted KEGG (Kyoto Encyclopedia of Genes and Genomes) enrichment analysis to identify pathways regulated by viral miRNAs to provide biological insights. KEGG pathway is a collection of manually drawn pathway maps representing molecular interactions and reaction networks, and has been widely used for biological interpretation of higher level systemic functions [19]. KEGG enrichment analysis is calculated by R package SubpathwayMiner [20], which implements hypergeometric test to measure *p*-value for evaluating enrichment significance of pathways. SubpathwayMiner also provides the FDR-corrected *q*-values to reduce the false positive discovery rate [20].

### Comparing viral miRNA regulated pathways

Significant KEGG pathways regulated by different viruses were compared and visualized using our in-house developed R package clusterProfiler http://bioconductor.org/packages/2.8/bioc/html/clusterProfiler.html. ClusterProfiler, which was implemented based on R and its plotting system ggplot2 [21], is released under the Artistic-2.0 license within Bioconductor project [22]. ClusterProfiler was designed to provide statistical analysis of GO and KEGG and visualization tools for comparing functional profiles among gene clusters. More details on the use of clusterProfiler are available in the package vignette.

### Phylogenetic analysis

We built phylogenetic trees of human viruses based on the functions their miRNAs encoded. Phylogenetic trees were constructed by R package phangorn [23] using the popular neighbour-joining (NJ) method. For validating our phylogenetic analysis, we compared our results with phylogenetic trees obtained from whole genome sequence alignment. Complete genome sequences of viruses were obtained from NCBI nucleotide database. Multiple sequence alignment and phylogenetic tree construction were done by ClustalX (version 2.0.12) [24] using NJ algorithm. Robinson-Foulds (RF) metric [25], the most widely used method in comparing phylogenetic trees, was adopted to compute the topological distance between phylogenetic trees. RF rate was obtained by normalizing the RF distances by the number of total edges for representing the relationship among trees [26]. RF rate measures the dissimilarity between two trees.

## Results and Discussion

**Figure 1:**
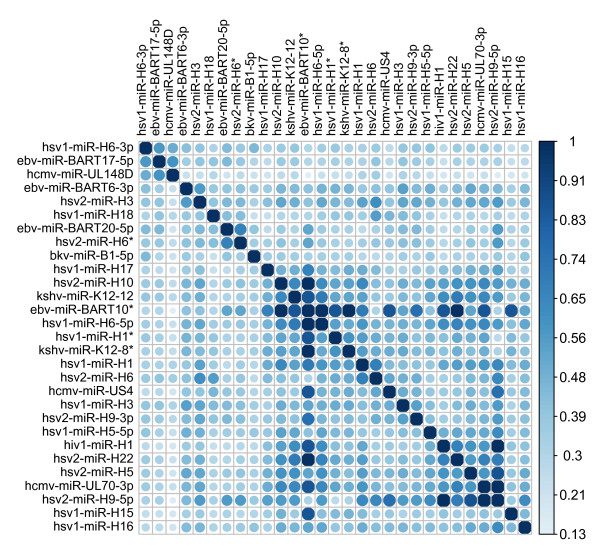
**Functional similarity matrix of viral miRNAs**.

We applied our method [14] to assess similarities among viral miRNAs. As a result, we obtained the pairwise functional similarity of 29 viral miRNAs as illustrated in Figure 1.

**Figure 2:**
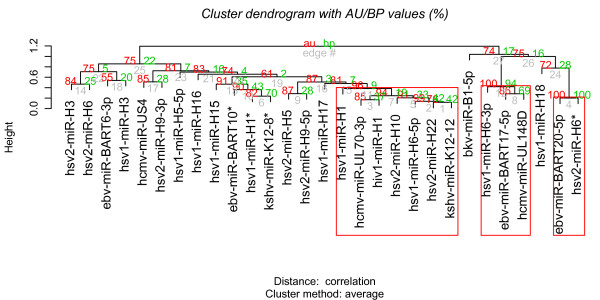
**Hierarchical clustering viral miRNAs with *p*-values**.

The functional similarity matrix of the pairwise viral miRNAs was then analyzed by R package pvclust to assess the uncertainty of clustering result [17]. We obtained 3 clusters with AU *p*-value > 0.95. These 3 clusters contain 2 (ebv-miR-BART20-5p and hsv2-miR-H6*), 7 (hcmv-miR-UL70-3p, hiv1-miR-H1, hsv1-miR-H1, hsv1-miR-H6-5p, hsv2-miR-H10, hsv2-miR-H22 and kshv-miR-K12-12), and 3 (ebv-miR-BART17-5p, hcmv-miR-UL148D and hsv1-miR-H6-3p) miRNAs as illustrated in Figure 2.

GO enrichment analysis was performed across these three significant clusters to discover their biological themes. As a result, Cluster 1 suggests the down-regulation of xylosyltransferase activity, involved in O-glycan processing. O-glycans had been described to play roles in cell polarity [27], which involves in the formation of immunological synapse [28], indicating that viruses prevent the formation of immunological synapse by inhibiting the xylosyltransferase activity. Cluster 2 represses a wide range of binding activities, including protein binding, DNA binding, receptor binding, and enzyme binding. Especially, the inhibition of MHC protein binding and CD40 receptor binding suggests that viruses use miRNAs to interfere the activation of antigen presenting cells. This may be the strategy for viruses to extend the life of the infected cells and to establish a favourable environment for their replication. Cluster 3 down-regulates transcription factor activity to favour viral latency. EBV BART miRNAs were expressed in latent infection [29]. Hsv1-miR-H6-3p had been reported to promote latency by inhibiting the expression of HSV-1-encoded transcription factor, ICP4, that is required for the expression of most HSV-1 genes during productive infection [30,31]. It has been reported that viruses encode proteins to interfere with transcription factors, and that miRNAs are more versatile to reshape the cellular status to escape host immune system and to hijack cellular machinery for their replication [32,33].

The average similarity among 29 viral miRNAs is only 0.434 and most of the miRNAs cannot be clustered with AU *p*-value > 0.95, indicating that a majority of these viral miRNAs have distinct functions, with the versatilities and flexibilities of viral regulations.

**Figure 3:**
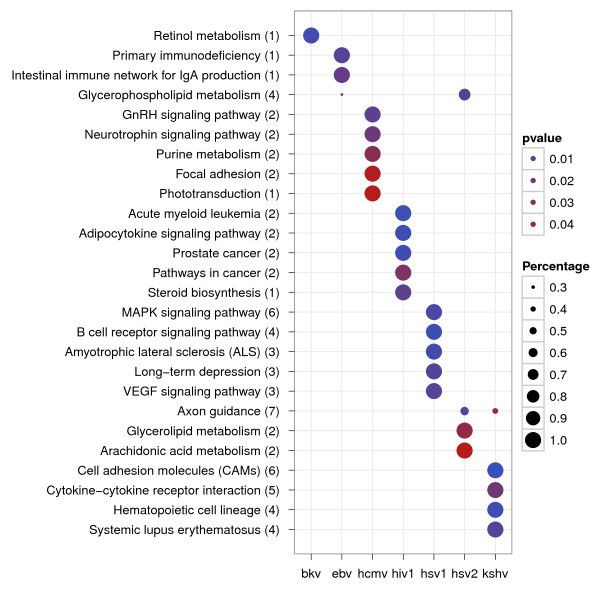
**Comparison of enriched pathways regulated by virus-encoded miRNAs**. The sizes of the dots represent the percentage of each row (KEGG category), and *p*-values were calculated by hypergeometric tests.

Viral infection generally results in dramatic alterations in cellular mRNA expression. We thus further identified cellular pathways perturbed by viral miRNAs using KEGG enrichment analysis to gain a higher level perception. The statistically and significantly enriched pathways perturbed by different viruses were then compared and illustrated in Figure 3.

As shown in Figure 3, different viruses have distinct strategies to reshape cellular status. It seems that viral miRNAs were designed to against many important pathways to favour their pathogenesis. KSHV-encoded miRNAs had been described to directly down regulate a major regulator of cell adhesion, THBS1 [34], that is involved in the recruitment of monocytes and T cells to the sites of infection [35]. Down regulation of THBS1 by KSHV miRNAs may aid KSHV-infected cells in avoiding detection by the host immune system [30]. HIV1-encoded miRNAs play critical roles in oncogenic transformation [36], and three miRNAs encoded by EBV are crucial for efficient B cell transformation [37]. These biological findings are consistent with our analyses. In addition, many pathways in our analyses have not been reported yet, and thus can serve as putative functions played by viral miRNAs for further investigations.

**Figure 4:**
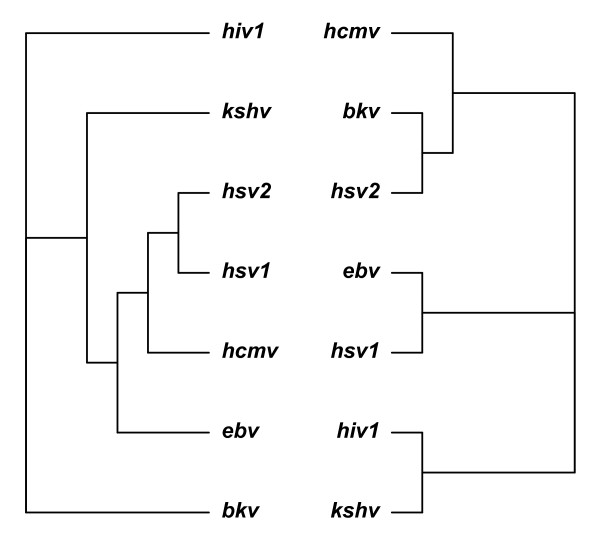
**Phylogenetic trees of human viruses, constructed from genome sequence alignment (left) and functional similarity of viral miRNAs (right)**.

Reconstructing the tree of virus phylogeny is still the cardinal challenges in biology. Here we used the similarity index by functions that viral miRNAs encoded to rebuild the phylogenetic tree. We then compared our tree with phylogenetic tree obtained by genome alignment as shown in Figure 4. Although the tree based on genome alignment included biases like horizontal gene transfer (HGT) [38], genome alignment is still the *de facto *standard for phylogenetic tree construction.

We evaluated the similarity between these two trees. The topological distance between them was calculated by RF metric to be 8, and the corresponding RF rate is 0.727, and thus the similarity between the two trees is 0.273. Surprisingly, viral miRNAs have distinct evolution rates compared to their corresponding genome based on our functional analysis. We thus measured the evolutionary distance among viruses by their encoded miRNA sequences. RF distance between phylogenetic trees obtained from genome sequences and miRNA sequences is 6, and the corresponding RF rate is 0.545, and thus the similarity between the two trees is 0.455.

Viral miRNAs have different properties compared with viral proteins, such as small and non-immunogenic, and thus they may serve as ideal tools to interpolate cellular environment in the ways that benefit virus replication. This would mean an evolutionary reward for rapid adjustment to the host and environmental statuses. Viral miRNAs do not share a high level of homology even within the members of the same family [6,39]. Phylogenetic analysis of all previously known virus miRNA genes showed that most of the known viral miRNAs have long distant relationships and could be classified into specific miRNA families [40]. These findings are consistent with our phylogenetic analysis, suggesting that viral miRNAs may evolve more rapidly than their genome. Especially, the functions of viral miRNAs evolve even more rapidly than their sequences.

Obviously, miRNAs are ideal for the tight space constraints characteristic of viral genomes and the evolution of a miRNA down-regulating a new target gene can presumably be achieved more easily than the evolution of a new protein [30]. It must be pointed out, however, our current method only provides a perception of viral miRNA perspective and may contain some biases, as it did not consider the fact that the activation of viral miRNAs depends on the viral life cycle in various latent or at lytic stages, and the specific infected cell types.

## Conclusions

Intimately connected with various kinds of diseases, viruses pose a crucial health problem on host. Host cellular expression profiles altered by virus-encoded miRNAs form a new regulatory layer. Though studies into pathogenesis by viral miRNAs are still in its infancy, the interspecies regulation at the miRNA level fuels the spark of the investigation into the repertoire of virus-host interactions. Here, we applied our method to assess the functional similarity among viral miRNAs. Our analyses showed that viral miRNAs have diverse functions. We then summarized cellular pathways regulated by viral miRNAs by the GO and KEGG enrichment analyses. Phylogenetic trees were reconstructed to reveal the evolutionary distance at the perspective of viral miRNAs.

Experimental validation of computational results is still a challenge, a hindrance towards understanding the functions of viral miRNAs. We believe that the integration of bioinformatics with microarray and proteomic data would be a promising way to elucidate the whole picture of virus-host interaction mediated by viral miRNAs. In addition, the identification of roles played by viral miRNAs in pathogenesis would help in designing new preventive and therapeutic approaches. This has also been described as new therapeutics to correct the aberrant activity of miRNA-mRNA interaction by using anti-miRNA oligonucleotides (AMOs) [41]. We hope that this work can provide a better understanding of basic biological processes involved in latency and oncogenic transformation mediated by viral miRNAs.

## Competing interests

The authors declare that they have no competing interests.

## Authors' contributions

G Yu conceived and designed the prototype of the study, conducted the data analyses and drafted the manuscript. QY He supervised the study. All authors approved the final manuscript.
